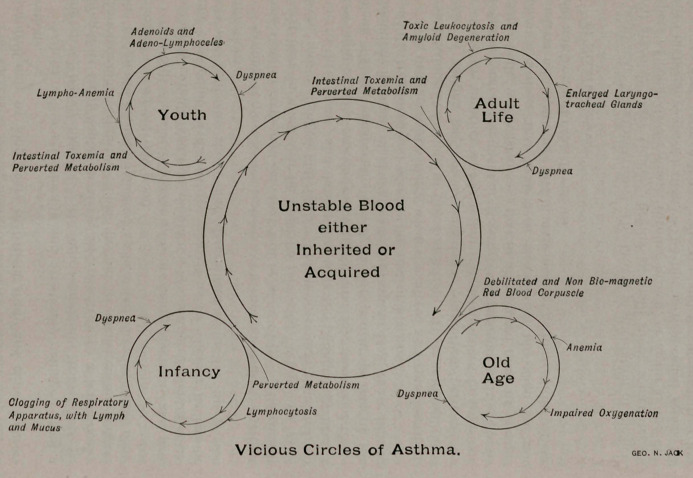# The Prognosis of Asthma from a Digestive Blood Metabolic Etiologic Standpoint1Read at the quarterly meeting of the Erie County Medical Association, June 12, 1905.

**Published:** 1905-08

**Authors:** George N. Jack

**Affiliations:** Buffalo, N. Y.; 91 Niagara Street


### The Prognosis of Asthma From a Digestive Blood Metabolic Etiologic Standpoint.[1]

By GEORGE N. JACK, M. D., Buffalo, N. Y.

IN PREVIOUS papers I have demonstrated quite satisfactorily in the opinion of some of our best pathologists, our most acute diagnosticians and our most eminent therapeutists that asthma is a digestive, blood, metabolic, disease and not a neurotic, spasmodic or turgescent one as heretofore maintained. Is it surprising then that not knowing the etiology of asthma its prognosis should be unsatisfactory? Thus we find in every textbook that attempts to give a prognosis a very unfavorable and discouraging one to both physician and patient. As regards the prognosis of asthma, Stevens says, "The disease does not prove fatal except through complication or sequelæ. In young persons without an inherited tendency the prognosis should be guardedly favorable. The older the patient the greater the inherited tendency, the more unfavorable becomes the prognosis."

Salter has a still more unfavorable prognosis. He lays much stress on the age, stating that "Above forty-five the recoveries are few indeed; the tendency is generally toward a progressive severity of the disease and the prognosis should always be guarded, if not absolutely unfavorable."

This prognosis does not spur the physician on to cure his patient, neither is it very pleasant for the asthmatic to consider. To illustrate the discouraging prognosis that most physicians have for asthma I will quote an incident that occurred to me when I first began to make a specialty of treating asthma.

One of my old college professors met me in an instrument store and he remarked. "I see that you intend making a specialty of asthma." I answered in the affirmative. "Well," he replied, "any one who succeeds in curing asthma will become a millionaire."

Since then I have treated many cases of asthma, none of which, barring the complicated cases, are now wheezing, but I am as yet far from being a millionaire.

The sentiments and failures of the profession concerning asthma have been absorbed and recorded by the laity.

The vast majority of my patients that have not been referred to me by other physicians, come tentatively and doubtfully. They all have the same story. They have paid out vast sums of money "doctoring" with no benefit. They have suffered all the unde-

---

1. Read at the quarterly meeting of the Erie County Medical Association, June 12, 1905.

scribable agonies of the disease, with the mockery of their would-be consoling friends and relatives who assure them that they will not die, as no one ever dies with asthma, while inwardly this is a characteristic of the disease that they most sadly regret. They do not believe that there is any permanent relief for asthma nor that "doctoring" does any good.

It is no wonder that asthmatics' experience with physicians has been discouraging owing to the physician's mistaken etiology. It would be as reasonable to attempt to clear out a stove pipe clogged with soot by burning antispasmodics in the stove as to attempt to relieve an asthmatic by administering antispasmodics. The prognosis of asthma when scientifically managed from a digestive, blood, metabolic, etiologic standpoint assumes an entirely different aspect than when treated from the ancient and now existing textbook neurospasmodic etiologic conception. From an unfavorable, doubtful and guarded prognosis it becomes a positively favorable one.

There is at present and through all time has been so much uncertainty in the management of chronic diseases, that when one feels his feet resting on a rock he stands there with pride and determination that no absurd or mistaken theories can swerve. Scientific investigation, clinical research and accurate mathematical reasoning have finally succeeded in placing the management of asthma on a rock bottom scientific foundation.

This conquest has increased the physician's usefulness by adding to his armamentarium a new but unfailing weapon with which to combat disease.

It enables us to make this surprising optimistic but, nevertheless, truthful prognosis: provided, that a careful scientific and accurate analysis has been made of the blood, urine, and stool, together with a thorough examination of all the viscera of the patient; these observations then to be verified or modified by a detailed clinical history of the patient. Now if the dietetic, hygienic, and medicinal treatment be adjusted to the demands and deficiencies of the case, as I have explained in previous papers, his functional equilibrium will be reëstablished and his asthma will disappear. Or, in other words, given an uncomplicated case of asthma, with sufficient financial means to provide the necessary medicines, together with a patient of sufficient intelligence and determination to faithfully follow out the hygienic and other directions, and the blood etiologic management will, with a mathematical certainty, produce in the beginning satisfactory results, and if persisted in, end in a permanent cure. In fact, many cases of asthma of years standing have never wheezed a wheeze after first beginning the treatment. The reason that we are enabled to make such a favorable prognosis for asthma is due to the fact that its pathology is largely functional.

The only structural pathologic changes associated with asthma are the enlarged glands in the air tubes and its sequelæ, as emphysema and a dilated right ventricle. That the pathology of asthma is functional and not structural is illustrated by the vicious circles of asthma, which will here again be presented for your consideration.

It will be observed, as here illustrated, that the pathology of asthma does not disturb the structural integrity, but that it is chiefly of functional origin. Having, then, the management of a disease with a functional pathology and enough vital force back of its functional pathology to establish a hasty recuperation, by removing the cause, nature will rapidly and satisfactorily establish a harmony of function that will relieve all the distressing symptoms.

The vital adaptability and recuperative forces of the asthmatic are very active and they respond quickly to the proper stimuli. The establishment of a harmony of function, however, with a disappearance of the dyspnea does not mean that our patient is permanently cured, but it simply means that we have been successful in selecting a hygienic, dietetic and medicinal treatment that will permit our patient to follow his vocation in life under still existing pathologic conditions until nature has time to completely recuperate and eradicate the lesions.

Nature may require from two to three years to bring about these happy results, and during that time the patient should be under the constant supervision of a physician.

Throughout the whole course of treatment the patient's blood, urine, and stool should be regularly analysed and the dietetic, hygienic, and medicinal treatment adjusted from time to time according to these laboratory investigations. To illustrate the importance of these frequent laboratory investigations I will quote you a few rules which have proven essential to the successful management of asthma.

First.—Physiologic measures are when possible to be substituted for drugs.

Second.—Blood showing an excess of lymphocytes indicates the withholding of lymphogenous foods, as milk and raw oysters.

Third.—Blood giving a pronounced iodophilia indicates the withholding of starch foods.

Fourth.—Blood showing a lack of fibrin indicates the administering of gelatine.

Fifth.—A toxic cadaveric stool indicates the withholding of proteids or meats.

Sixth.—Urine containing indican indicates an intestinal toxemia and a restricted diet.

There are hundreds of other things to be determined by lab-

Vicious Circles of Asthma.

oratory investigation which time will not permit mentioning. Then, again, the experienced eye will obtain much valuable data as to the physiologic adaptability of the patient to the prescribed treatment by frequent observations as water bags under the eye, or swollen fingers, denoting stagnated lymph; coated tongue or flatulency, denoting a poorly adjusted diet, and the like.

The successful management of asthma is an art that can be acquired only by long years of patient study and clinical observations.

Asthma, when scientifically and artistically managed from a digestive, blood, metabolic, etiologic standpoint transforms its prognosis from doubt, mystery, and obscurity to a scientifically demonstrable certainty. This method of management renders the prognosis of uncomplicated asthma and likewise many complicated cases positively good.

The only element of doubt as to its being positively and permanently curable, and that should not be considered from a scientific standpoint, is the patient himself as to whether he has or has not sufficient ability and determination to live up to the directions.

Asthma, it must be remembered, is a chronic disease; one that was slow to come and one that will be slow to go. The patient must bear in mind that he must be remolded or regrown, as it were, into a changed individual. This process may require from one to three years, and during that time the patient may have a few light attacks, especially during the first few months of treatment. These exasperations will, if our patient is one of the doubting, ignorant class that cannot grasp an idea, cause him to quit the treatment and wheeze the rest of his life.

The prognosis of asthma, when considered as above described, can be simmered down to this conclusion. If an uncomplicated case of asthma has not its functional equilibrium permanently restored or, in other words, is not completely cured in from one to three years' treatment it is either the patient's or the physician's fault, which is to say, that in all uncured cases of uncomplicated asthma that have been treated the physician did not understand the etiology and management of asthma or the patient did not live up to the directions.

91 Niagara Street.

---

Arsenical Pigmentation.—The brown pigmentation of medicinal arsenism, says Gowers, begins as small spots, sometimes as spots of congestive redness, turning brown, or again as the diffuse mahogany discoloration. The tint is that called sepia. It is not so dark as in Addison's disease, and the pigmentation has not the same distribution, being most marked at areas subjected to pressure. When the small, discrete spots coalesce, they often leave small unpigmented areas of pearly whiteness.—*Denver Med. Times.*

## Figures and Tables

**Figure f1:**